# MicroRNAs in Atrial Fibrillation: from Expression Signatures to Functional Implications

**DOI:** 10.1007/s10557-017-6736-z

**Published:** 2017-07-28

**Authors:** Nicoline W. E. van den Berg, Makiri Kawasaki, Wouter R. Berger, Jolien Neefs, Eva Meulendijks, Anke J. Tijsen, Joris R. de Groot

**Affiliations:** 1 Department of Cardiology, Heart Center, Academic Medical Center/University of Amsterdam, Amsterdam, The Netherlands; 2 Department of Experimental Cardiology, Heart Center, Academic Medical Center/University of Amsterdam, Amsterdam, The Netherlands

**Keywords:** Atrial fibrillation, microRNA, Fibrosis, Electrical remodelling, Therapy

## Abstract

Atrial fibrillation (AF) is the most common sustained arrhythmia and is associated with pronounced morbidity and mortality. Its prevalence, expected to further increase for the forthcoming years, and associated frequent hospitalizations turn AF into a major health problem. Structural and electrical atrial remodelling underlie the substrate for AF, but the exact mechanisms driving this remodelling remain incompletely understood. Recent studies have shown that microRNAs (miRNA), short non-coding RNAs that regulate gene expression, may be involved in the pathophysiology of AF. MiRNAs have been implicated in AF-induced ion channel remodelling and fibrosis. MiRNAs could therefore provide insight into AF pathophysiology or become novel targets for therapy with miRNA mimics or anti-miRNAs. Moreover, circulating miRNAs have been suggested as a new class of diagnostic and prognostic biomarkers of AF. However, the origin and function of miRNAs in tissue and plasma frequently remain unknown and studies investigating the role of miRNAs in AF vary in design and focus and even present contradicting results. Here, we provide a systematic review of the available clinical and functional studies investigating the tissue and plasma miRNAs in AF and will thereafter discuss the potential of miRNAs as biomarkers or novel therapeutic targets in AF.

**Electronic supplementary material:**

The online version of this article (doi:10.1007/s10557-017-6736-z) contains supplementary material, which is available to authorized users.

## Introduction

Atrial fibrillation (AF) is the most common sustained arrhythmia, associated with a pronounced morbidity and mortality. AF is a major healthcare problem as it possesses a serious burden on socioeconomic budget due to its increasing prevalence and associated frequent hospitalizations. The most important risk factor for new-onset AF is ageing, and ageing of the population importantly contributes to the growing number of patients with AF. AF prevalence is expected to double by 2050 [1, 2]. The disease may present as lone AF or in association with other systemic or cardiovascular diseases such as hypertension, obesity, ischemic heart disease, or valvular disease [2, 3].

AF is a progressive disease that is classified on the basis of clinical presentation, duration and spontaneous termination of AF episodes. AF is subsequently categorized as ‘paroxysmal AF’ (parAF), defined by self-terminating or cardioverted AF within 7 days after onset, ‘persistent AF’ (persAF), defined by AF lasting longer than 7 days, ‘long-standing persAF’, which is AF lasting over 1 year and ‘permanent AF’ (permAF), when patient and physician have accepted that the rhythm will not return to normal sinus rhythm [4]. Recently, the concept of ‘atrial cardiomyopathy’ was introduced, which proposes a classification based on the pathological processes present in atrial disease in addition to the clinical classification [5]. However, we are insufficiently able to recognize the electrical and structural remodelling underlying the atrial cardiomyopathy present in AF patients. Moreover, AF remodelling may differ per aetiology or comorbidities present and does not necessarily parallel to the staged clinical classification [6]. Not surprisingly, treatment strategies that target atrial remodelling are lacking [4, 7]. A better understanding of the pathophysiological processes underlying AF and non-invasive methods to classify patients based on the underlying atrial cardiomyopathy would enable early and mechanism-specific diagnosis of AF followed by the development of mechanism-specific treatments [8].

Emerging studies have uncovered a role for microRNAs (miRNA, miR) in the initiation and maintenance of cardiovascular disease [9]. MiRNAs are highly conserved, small, non-coding RNAs that regulate physiological and disease processes at the post-transcriptional level. MiRNAs bind to a (partially) complementary sequence in the 3′-untranslated region (3’UTR) of the mRNA and can thereby induce degradation or inhibition of translation of this mRNA [10]. MiRNAs regulate approximately 30% of all protein-coding genes and have therefore been predicted to be involved in almost all cellular processes [11].

MiRNA regulation in cardiovascular disease was first described in 2006, when specific miRNAs were found to be up- or downregulated in mouse models of cardiac hypertrophy and heart failure (HF) [9]. Later, it was demonstrated that AF is associated with altered miRNA levels in atrial tissue and plasma [12, 13]. The regulatory function of specific miRNAs has been studied in the structural and electrical remodelling underlying AF, ischemic heart disease, cardiac hypertrophy, ion channel modification and extracellular matrix (ECM) formation [9, 14–19]. Furthermore, circulating miRNAs associated with AF could serve as potential biomarkers of the disease, whereas specific tissue miRNAs could become targets for therapy [20–22].

Currently available studies investigating the role of miRNAs in AF are diverse, with various designs and focuses, and show contradicting results. This systematic review aims to provide a structured overview of the available clinical studies exploring the tissue and plasma miRNA expression profiles in AF patients. Next, we will discuss the available experimental evidence for the functional role of miRNAs in pathophysiological processes underlying AF and discuss the future possible clinical applications of miRNAs in AF.

## MiRNAS as Biomarkers of Atrial Fibrillation

MiRNAs may be suitable as biomarkers of disease, because of their tissue- and pathology-specific expression. They are stable in plasma because they are either incorporated in microparticles (exosomes, microvesicles and apoptotic bodies), or bound to proteins or high-density lipoproteins and are thereby protected from RNase activity. Moreover, miRNAs are detectable in plasma or serum with high sensitivity and specificity [20, 23, 24].

MiRNAs have been suggested as biomarkers of several cardiac diseases, including heart failure and coronary artery disease [25–27]. For example, plasma miR-208b demonstrated a high diagnostic accuracy for myocardial infarction similar to troponine T [28, 29]. This example underscores the potential of miRNAs to serve as clinical biomarkers of cardiovascular disease, while no such biomarker is currently available for AF. Once a diagnosis of AF has been established, biomarkers may give insight into the specific atrial cardiomyopathy underlying AF, which may have implications for prognosis and treatment and may enhance patient-tailored care [4].

### MiRNAs Associated with Atrial Fibrillation Onset

McManus et al. [30] performed the only study to date that evaluated the prognostic value of circulating miRNAs for the occurrence of new-onset AF. In this study, which included 2292 participants from the Framingham Heart cohort without AF, 107 participants developed AF after a median follow-up of 5.4 years, but none of the 385 investigated miRNAs in whole blood were associated with new-onset AF.

Two prospective studies [31–34] investigated whether the occurrence of postoperative AF (POAF) was associated with miRNA plasma levels at the time of coronary bypass surgery (CABG) (Online Resource Table 2). POAF has a complex, but specific aetiology that involves considerable systemic and local inflammation following cardiac surgery [35]. Harling et al. [31] collected serum prior to surgery and concomitantly retrieved atrial tissue during cardiac surgery. They found 16 miRNAs that were differentially expressed in atrial tissue between 11 patients who developed POAF and 11 patients who did not. The cardiomyocyte-enriched miR-208a (further described in Tables 3 and 4) was most downregulated and miR-483-5p most upregulated. MiR-483-5p serum levels were also increased in these patients (ROC area 0.78) whereas miR-208a was undetectable in serum at any time point. Krogstad et al. [34] used qPCR to study the levels of over 30 miRNAs in plasma of 92 CABG patients, of whom 27 developed POAF. However, they found no miRNA associated with POAF onset.

Hence, the prognostic value of individual miRNAs has not been irrevocably demonstrated. MiR-483-5p was the only circulating miRNA associated with the occurrence of POAF and no circulating miRNA was associated with new-onset AF. Tissue miRNAs were more frequently associated with POAF than circulating miRNAs. However, as tissue is not standardly retrieved during (cardiothoracic) surgery, standardization of retrieval and processing is needed before tissue miRNAs can be considered for the prognostication of (PO)AF. Altogether, there is insufficient evidence to implement current findings into clinical practice. Future studies investigating the prognostic value of miRNAs for new-onset AF or POAF should focus on high risk patients and perform extensive clinical profiling to enable differentiating patients according to AF aetiology.

### Circulating miRNAs Associated with Prevalent Atrial Fibrillation

**Fig. 1:**
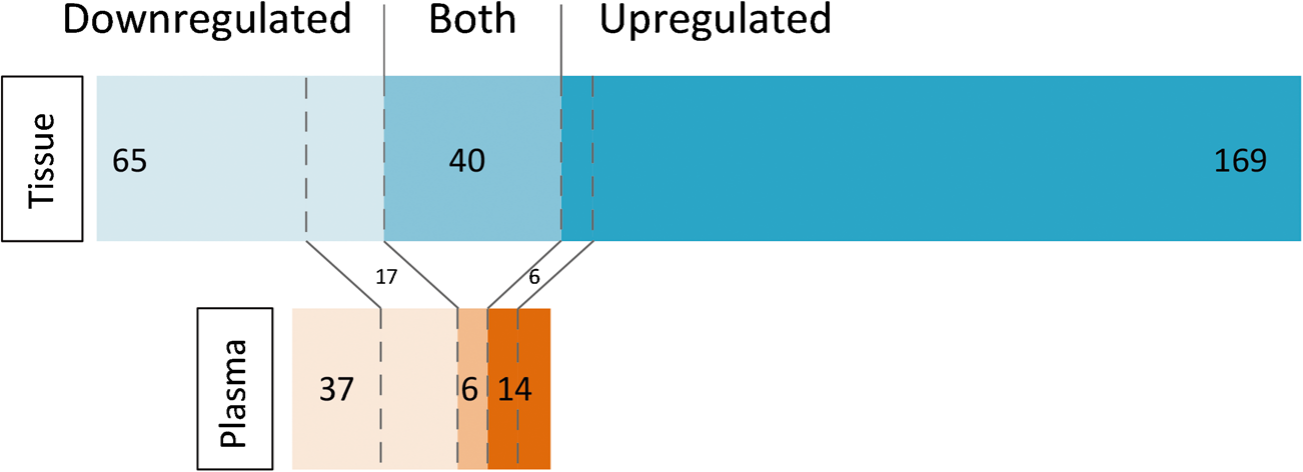
microRNAs expressed in plasma and tissue. This figure illustrates the number of miRNAs differentially expressed in tissue and in plasma in AF patients. Note the high number of upregulated miRNAs in tissue, whereas miRNAs in plasma more often have lower levels in AF. Furthermore, there is little overlap between tissue and plasma expression of the miRNAs that have lower or higher levels in AF (between the dotted lines). There is a substantial proportion of miRNAs that present contradicting results (miRNAs described to be both up- and downregulated in tissue or plasma)

We identified 6 studies [30, 36–39] investigating miRNAs in plasma samples from patients with AF and controls without AF (Table 1). Twenty circulating miRNAs demonstrated higher levels in AF patients versus controls in one or more studies and 43 miRNAs demonstrated lower levels. (Fig. 1. Online Resource Table 3 gives a complete list of plasma miRNAs in AF)

**Table 1:** MicroRNA discovery studies in plasma

Ref	Study population	Technique	miRNA expression in AF patients
35	HF patients with EF <40% and healthy volunteers: 15 HF and AF 26 HF no AF 35 matched controls	Platelets: microarray Serum: qPCR of 89 miRNAs	Upregulated: None Downregulated: miR-150 (both plasma and platelets)
36	Discovery phase: 5 parAF, 5 persAF, 5 controls Validation phase: 30 parAF, 30 persAF, 30 controls	MPSS qPCR (146a, 150, 19a, 375)	Upregulated: 19a, 125a-5p, 146a, 146b-5p, 148b, 221, 342-3p, 409-3p, 421, 589, 598, 941 Downregulated: 99b, 100, 150^b^, 199a-5p, 199b-5p, 320b, 375
37	Discovery phase: pooled samples 30 AF, 30 controls qPCR validation: pooled independent samples 30 AF, 30 controls qPCR validation: non-pooled independent samples 40 AF, 40 controls.	Solexa sequencing qPCR	Upregulated: 9, 152, 374a, 454, 664 Downregulated: 16–2^a^, 328^b^, 338-5p, 409-3p^b^, 432^b^, 478b, 486-5p, 493, 766, 874, 4732-3p
29	153 prevalent AF at baseline 1017 new-onset AF after median FU 5.4y 2185 No AF at baseline or FU	qPCR of 385 miRNAs	Upregulated prevalent AF: 31-3p, 182-5p, 196b-5p Downregulated prevalent AF: 28-5p, 99b-5p, 150-5p, 328, 331-3p, 339-5p Upregulated incident AF:29b-2-5p, 134, 151a-3p, 152, 193a-5p, 200c-3p, 221-3p, 375, 1274b, 720 Downregulated incident AF: None
39	112 AF 99 no AF	High throughput qPCR of 86 miRNAs LAA removed	Upregulated: none Downregulated: let-7b-5p, let-7c-5p, 10b-5p, 21-5p, 24-3p, 29a-3p, 30c-5p, 99b-5p, 100-5p, 122-5p, 125a-5p, 125b-5p, 126-3p, 146a-5p, 148b-3p, 150-5p, 221-3p, 223-3p, 342-3p, 375, 411-5p
38	122 AF (31 parAF, 91 pers. + permAF) 122 no AF	Microarray (4 pooled groups)	Upregulated: 9, 19, 146, 152,374a, 454, 634, 664 Downregulated: 1, 145, 162, 222, 328, 432, 493b

Abbreviations: *AF* atrial fibrillation, *EF* ventricular ejection fraction, *FU* follow-up, *HF* heart failure, *LAA* left atrial appendage, *MPSS* massively parallel signature sequencing, *parAF* paroxysmal AF, *permAF* permanent/chronic AF, *persAF* persistent AF, *qPCR* quantitative polymerase chain reaction

^a^Anti-sense miRNA

^b^Validated with qPCR

^c^Most upregulated or downregulated miRNA

A limited number of miRNAs were consistently reported to have higher plasma levels in AF compared to controls. MiRNAs with higher levels in AF patients in two studies were miR-9, miR-152, miR-374a, miR-454 and miR-664. No miRNA was reported to be increased by three or more studies. More often, lower miRNA levels in AF were seen. MiRNAs with lower levels in AF described by three or more studies were miR-99b, miR-150 and miR-328. Interestingly, these three miRNAs were also reported by McManus et al. [30], who studied new-onset AF in the Framingham Heart cohort and used the same cohort to compare 2185 participants without AF with 153 patients with prevalent AF. In this study, miR-99b, miR-150 and miR-328 showed lower levels in AF, but only miR-328 levels remained significantly lower in patients with prevalent AF after correction for age, sex and technical covariates, like RNA quality and concentration.

MiR-150 was lower in plasma of AF patients in 4 out of 6 miRNA discovery studies [30, 36, 39, 40]. Goren et al. [36] included 41 patients with heart failure (HF), with or without AF, and found decreased levels of miR-150 in both plasma and platelets of AF patients. After a full separation of platelets and plasma, miR-150 was abundantly present in platelets. Platelet and plasma levels were significantly correlated, suggesting that platelets are the origin or transport mode of miR-150 [36]. The exact functional role of miR-150 in AF pathophysiology was not established by this study. Goren et al. [36] speculated that lower platelet miR-150 could be involved in many pathways leading to AF, including inflammation, platelet function and fibrosis [36, 41, 42]. Another study demonstrated that miR-150 promotes megakaryocytopoiesis in platelet progenitor cells [43].

Some of the miRNAs reported in explorative plasma studies were also described by studies exploring atrial tissue miRNA expression. For example, miR-99b was found to be decreased in plasma studies [30, 37, 40] and also in the left atrial appendage (LAA) of AF patients [44]. Overall, however, there was limited correlation between the levels of miRNAs discovered in plasma and tissue levels. For instance, miR-150 and miR-328, for which there is considerable evidence for lower plasma levels in AF patients, have not been described by any explorative study investigating atrial tissue. Conversely, miR-328 was actually upregulated in a study using a canine tachypacing model [45]. Interestingly, overall a larger proportion of miRNAs was found to be decreased in AF in plasma, whereas most differentially expressed miRNAs in tissue were found to be upregulated (Fig. 1).These findings illustrate that the differential expression in tissue does not necessarily give rise to different miRNA levels in plasma, and vice versa. However, higher miRNA levels in tissue and inverse lower levels in plasma suggest that tissue overexpression may at least partly result from an active process of miRNA retention in the cell or uptake of miRNAs from the circulation.

### Myocardial miRNAs Associated with Prevalent Atrial Fibrillation

We identified 12 original case-control studies (including at least four cases per study group) in 9 non-overlapping study populations, which explored miRNA expression in atrial tissue. Studies were performed between 2011 and 2016 and used a diverse range of microarray platforms and cut-offs for the discovery of differentially expressed miRNAs. In general, studies validated their most significant results by qPCR (Table 2).

**Table 2:** microRNA discovery studies in tissue

Ref	Tissue	Study Population	Technique	miRNA expression in AF patients
67	RAA	CABG, AVR and/or MVR: 4 parAF 4 matched no AF	mRNA-seq miRNA-microarray qPCR	Upregulated: 26a-2-3p, 27b-3p, 30b-3p, 30e-3p, 101-3p, 125a-5p, 125b-5p, 145-3p, 199a-3p, 199a-5p, 199b-5p, 222-3p, 223-3p, 1910-3p, 3135b, 3197, 381-5p, 3939, 4280, 4486, 6753-5p, 6820-5p, 7843-5p Downregulated: 1227-5p, 1273 g-3p, 1915-3p, 3196, 3656, 3665, 3960, 4281, 4466, 4497, 4516, 4530, 4690-5p, 4707-5p, 4734, 4787-5p, 4800-3p, 5096, 5787, 6090, 6125, 6775-5p, 6786-5p, 6791-5p, 7110-5p, 7704
43	MVR: RAA, LAA CABG: RAA HTx: LAA	~7 MVR and permAF ~6 MVR no AF 8 CABG no AF 5 HTx no AF	Microarray qPCR	Upregulated in RAA: 16, 21†, 21*, 142-3p, 142-5p, 146b-5p†‡, 198, 223, 224, 337-5p, 377, 483-5p, 1202, 1290, 1308 Downregulated in RAA: let-7c, 22*, 24–1*, 29c*,30‡, 30a, 30a*, 30b, 30c†, 30e, 99a, 99b, 125b-2*, 128, 133a†, 133b†, 139-5p, 143*, 145, 149, 181c, 181d, 197, 203, 331-3p, 367, 374b, 378, 378*, 484, 490-3p†‡, 490-5p, 628-5p No miRNAs were up- or downregulated in LAA.
70	RAA	CABG, AVR and/or MVR: 4 permAF 4 No AF	Microarray qPCR miR-499	Upregulated:1, 7–1*, 24–1*, 145*, 187*, 208b, 301a, 302a, 302b, 375, 454, 499†, 885-3p, 1244 Downregulated:21*, 23a*, 27a*, 138, 193b*, 299-3p, 1270
55, 45¥	LAA	6 MVR and AF 6 MVR no AF	Microarray qPCR	Upregulated: 15b-5p, 21-5p, 466†, 574-3p†, 3178, 3196, 3613-3p†, 4492, 4497, 4707-5p Downregulated: 1†, let-7 g-5p, 24-3p, 26a-5p†, 26b-5p, 29a-3p, 151a-5p, 195-5p, 361-5p, 720, 4454, 5100
	RAA, LAA	10 MVR and AF 8 MVR no AF	Microarray qPCR	Upregulated LAA: LAA: let-7d-3p, 15b-5p, 21-5p, 30a-5p, 149-3p, 181a-5p, 331-3p, 466, 494, 574-3p, 1307-3p, 1973, 3178, 3196, 3591-3p, 3613-3p, 3940-5p, 4485, 4492, 4497, 4534, 4707-5p Upregulated RAA: 149-3p, 574-5p, 762, 940, 1281, 1915-3p, 1973, 2861, 3141, 3656, 3940-5p, 4281, 4284, 4298, 4443, 4459, 4463, 4466, 4484†, 4485, 4488, 4497, 4505, 4508, 4530, 4534, 4707-5p, 4687-3p Downregulated LAA: let-7f-5p, 1†, 23b-3p†, 23c, 26a-5p†, 26b-5p, 143-3p†, 151a-5p, 151b, 195-5p, 361-5p, 378a-3p, 378d, 720, 2861, 4281, 4442, 4454†, 5100, 5190 Downregulated RAA: let-7a-5p, 16-5p, 21-5p, 22-3p, 25-3p, 26a-5p†, 26b-5p, 29a-3p, 30a-5p, 30b-5p, 30c-5p†, 30d-5p, 99a-5p, 107, 125a-5p, 125b-5p†, 133b†, 143-3p†, 145-5p†, 151a-5p, 151b, 152, 181a-5p, 191-5p, 195-5p, 221-3p, 222-3p, 331-3p, 378a-3p, 378d, 451a, 455-3p, 486-5p, 4286, 4324, 4454†, 5100
53	RAA	CABG, aortic (valve) repair, MVR, maze, TVR, septal defects 9 persAF 11 no AF	Microarray qPCR of miR-30 family	Upregulated: 22, 24, 24–1*, 30a, 30b*†, 30d*†‡, 30e, 125a-3p, 185, 208b, 210, 324-5p, 499-5p‡, 505*, 574-5p, 602, 652, 671-5p, 1181, 1224-5p, 1290, 1305, 1972, 1973, 3125, 3195, 3610, 3648, 3679-5p, 4257, 4291, 4298, 4299, 4306 Downregulated: 10a, 31, 100
52	RAA, plasma	CABG, aortic (valve) repair, MVR, TVR: 16 AF (4 permAF; 12 maze; 10 succesful maze) 13 no AF (CABG/AVR)	Microarray	Upregulated: let-7a, let-7d, let-7f, 20b, 21†‡, 22, 23b‡, 24, 27a, 27b, 28-5p, 32, 34a, 93, 95, 101, 103, 106b, 125b, 127-3p, 129-3p, 130a, 130b, 134, 140-5p, 142-3p, 146b,148b, 152, 15a, 15b, 181a, 181c, 184, 185, 187, 190, 193a-3p, 196b, 199a-5p, 199b-5p‡, 203, 208b†‡, 210, 215, 216a, 216b, 217, 320, 324-5p, 330-3p, 337-5p, 361-5p, 362-5p, 371-3p, 372, 423-5p, 424, 439, 449a, 450a, 455-5p, 487a, 487b, 494, 495, 499-5p, 500, 504, 505, 508-3p, 509-5p, 511, 517a, 517c, 518b, 518f, 520e, 522, 539, 542-5p, 545, 548d-5p, 579, 597, 618, 652, 660, 671-3p, 758, 874, 886-5p, 887, 888 Downregulated: 31, 200b, 429, 885-5p
107, 99¥	RAA, LAA	MVR, CABG and/or AVR: 21 AF (11 parAF; 10 permAF) 16 no AF	Microarray qPCR validation of all selected miRNAs Mitochondrial respiration	Upregulated: LAA: 18a, 18b, 19a, 19b, 23a, 25, 30a, 93, 106a, 106b, 144, 363, 451, 486-5p, 590-5p RAA: 15b, 106b, 144, 451 Downregulated in both LAA and RAA: 208a
102, 101	LAA	parAF, HTx: Discovery phase: 8 parAF; 5 HTx Validation phase: 30 parAF, 17HTx	Microarray qPCR	Upregulated: 19b†, 142-3p, 146b-5p†‡, 155†‡, 193b, 223, 301b, 486-5p, 519b-3p Downregulated: 193a-5p
68	RAA	9 MVR and AF 4 MVR no AF 9 HTx	Microarray qPCR	Upregulated: 188-5p, 212, 335†, 630,1181, 1202†, 1207-5p, 1225-5p Downregulated: 95, 26b*, 125a-5p, 125b, 125b-2*, 143*, 145, 145*, 149, 181a†, 181a*, 181b, 181c†, 181d, 324-5p, 497, 500, 501-5p, 550, 874

* anti-sense miRNA; † validated with qPCR; ‡ most upregulated or downregulated miRNA

¥ There is an overlap of study population between the 2 studies. The first reference focused on the comparison between AF and no AF whereas the second reference focused on a comparison between RAA and LAA.

Both studies by Wang *et al.* describe the exact same study population and microarray results

Abbreviations: *AF* atrial fibrillation, *AVR* aortic valve repair, *CABG* coronary artery bypass grafting, *HTx* heart transplantation, *LAA* left atrial appendage, *MVR* mitral valve repair, *parAF* paroxysmal AF, *permAF* permanent/chronic AF, *persAF* persistent AF, *RAA* right atrial appendage, *seq* sequencing, *qPCR* quantitative polymerase chain reaction

In total, 209 miRNAs were reported upregulated in AF in one or more studies and 105 miRNAs were downregulated. MiRNAs that were consistently upregulated in three or more studies were miR-15b, miR-21, miR-24, miR-30a, miR-142-3p, miR-146b, miR-208b, miR-223 and miR-499. Downregulated miRNAs described by at least three studies were miR-125b, miR-143 and miR-145. However, discrepancy exists concerning the up- or downregulation of five of these miRNAs (miR-21, miR-24, miR-30a, miR-125b, miR-145) (Online Resource Table 4 gives a complete list of tissue miRNAs in AF). The various origins of the tissue may in part explain this as exemplified by miR-21, which was downregulated in right atrial (RA) tissue, but upregulated in left atrial (LA) tissue [46]. However, tissue origin may not be the only explanation and an influence of study populations, comorbidities and techniques cannot be excluded.

## Functional Implications of Tissue MiRNAS in Atrial Fibrillation

A predisposition to AF results from atrial remodelling processes that are generally thought to involve ion channel remodelling, Ca^2+^ overload, structural remodelling such as fibrosis and autonomic dysregulation (extensively reviewed earlier [41, 47–50]).

Numerous explorative studies have implicated miRNAs in these AF-induced remodelling processes. These findings comprise association, but the functional targets are often only predicted by bioinformatics analysis and proof for an arrhythmogenic mechanism in AF is often lacking (Tables 1 and 2). Below, we discuss miRNAs described in functional studies that provide evidence for a specific regulatory role in AF by gain- and loss of function models and/or with luciferase reporter assays (Tables 3 and 4).

**Table 3:** MicroRNAs described for their role in electrical remodelling

miRNA in AF		Target(s) in AF		Experimental Model	Function	Reporting studies^a^
miR-31	↑	nNOS, dystrophine [52]	↓	51 AF, 165 SR patients, human cardiac myocytes, goat ATP model	Upregulation in AF correlated with decreased nNOS and unchanged Dystrophine mRNA levels. In vitro inhibition restored nNOS protein and normalized APD in myocytes from AF patients. MiR-31 directly targets both dystrophin and nNOS, and negatively reduce the respective protein by promoting nNOS mRNA decay and inhibiting the translation of dystrophin mRNA.	[30, 52–54]
miR-21	↑	CACNA1C, CACNB2 [59]	↓	10 permAF, 10 SR patients, atrial cadiomyocytes, HL-1 cells	An upregulationin AF was seen along with a decrease in CACNA1C, CACNB2 mRNA and I_CaL_ in human atrial cardiomyocytes. Its overexpression in vitro decreased I_CaL_ density and Cav1.2 protein levels. This miR directly targeted CACNA1C and CACNB2.	[40, 44, 46, 53, 56, 59, 74]
miR-208a/b	↑	CACNA1C, CACNB2 [58]	↓	16 permAF, 15 SR patients, sheep ATP, human cardiac myocytes, HL-1 cells	Upregulation of miR-208b, but not of miR-208a in AF patients compared to controls correlated with decreased mRNA, protein levels and I_CaL_ density. Overexpression of both miR208a/b in vitro reduced Cav1.2 protein levels. MiR-208a/b directly targeted CACNA1C and CACNB2.	[31, 53, 58, 62, 71, 74, 111, 114]
miR-328	↑	CACNA1C, CACNB1 [45]	↓	12 AF, 10 SR patients, canine ATP model, mice burst pacing, miR-328 TG mice, miR-328 sponge TG mice, in vivo forced expression in canines, neonatal rat cardiomyocytes	Upregulation in AF correlated with decreased human and canine mRNA and Cav1.2, Cavβ1 protein levels. In vivo overexpression of this miR promoted AF vulnerability, decreased APD, Cav1.2, Cavβ1 and I_CaL_density. Inhibition dampened AF vulnerability. MiR-328 directly targeted CACN1C and CACNB1.	[30, 37–39, 45, 56, 57]
miR-1	↑	KCNE1, KCNB2 [63]	↓	Rabbit ATP model, in vivo forced expression and inhibition in rabbits	ATP was associated with increased miR-1 decreased KCNE1 and KCNB2 mRNA and protein levels, shortening of AERP and an increase in IKs and AF susceptibility. MiR-1 in vivo overexpression further enhanced these effects, while inhibition with antimiR-1 alleviated these results. MiR-1 directly targeted KCNE1 and KCNB2.	[15, 46, 63, 74, 135]
	↓	KCNJ2 [15]	↑	31 AF, 31 SR patients, in vitro TP of human atrial slices	Downregulation in tissue of AF patients correlated with increased mRNA, Kir2.1 levels and I_K1_ density. TP of human atrial slices induced a miR-1 decrease and Kir2.1 increase.	
mir-26a/b	↓	KCNJ2 [16, 68]	↑	12 AF, 10 SR patients, canine A/VTP model, mice A/VTP model, in vivo forced expression in mice, canine and mice fibroblasts, TG and KO mice, H9c2 cells	Downregulation of miR-26b and miR-26a in particular in AF patients or AF animal models correlated with an upregulation of mRNA, Kir2.1 levels and I_K1_ density. Both in vivo and in vitro inhibition of miR-26 increased I_K1_ and AF vulnerability, whereas overexpression of dampened AF vulnerability. MiR-26 directly targeted KCNJ2.	[16, 46, 68–70, 86]
miR-30d	↑	KCNJ3 [71]	↓	14 AF, 19 SR patients, neonatal rat cardiomyocytes	Upregulation in cardiomyocytes from AF patients correlated with decreased mRNA and Kir3.1 levels. MiR-30d overexpression in vitro decreased KCNJ3, Kir3.1 and I_KACh_, while inhibition had the opposite effects. MiR-30d directly targeted KCNJ3.	[46, 71]
miR-499	↑	KCNN3 [74]	↓	4 permAF, 4 SR, HL-1 cells	Upregulation in tissue of AF patients correlated with decreased SK3 protein. MiR-499 in vitro overexpression suppressed KCNN3 levels and SK3 levels while inhibition enhanced SK3. MiR-499 directly targeted KCNN3.	[45, 53, 58, 71, 74]

^a^Studies reporting about the specific miRNA in AF. These include both explorative and functional studies in tissue and plasma and may present conflicting data regarding upregulation or downregulation of the miRNA

Abbreviations: *AF* atrial fibrillation, *APD* action potential duration, *ATP* atrial tachypacing, *cav1.2* L-type voltage-dependent calcium channel subunit α1C, *Cavβ1* L-type voltage-dependent calcium channel subunit β1, *I*_*Ca,L*_, L-type voltage-dependent calcium channel current, *KO* knockout, *LA* left atrium, *MVS* mitral valve stenosis, *ox-LDL* oxidized low-density lipoprotein, *permAF* permanent/chronic AF, *RA* right atrium, *SR* sinus rhythm/controls, *AERP* atrial effective refractory period, *AF* atrial fibrillation, *ATP* atrial tachypacing, *I*_*K1*_ inward rectifier K^+^ current, *I*_*KACh*_ acetylcholine regulated inward rectifier potassium current, *IKs* potassium currents, *Kir2.1* inward rectifier potassium channel 2, *Kir3.1* acetylcholine regulated inward rectifier potassium channel 3, *LA* left atrium, *permAF* permanent/chronic AF, *RA* right atrium, *SR* sinus rhythm/controls, *SK3* small conductance calcium-activated potassium channel 3, *TG* transgenic, *TP* tachypacing, *VTP* ventricular tachypacing

**Table 4:** MicroRNAs described for their role in structural remodelling

MiRNA in AF		Target(s) in AF		Experimental Model	Function	Reporting Studies^a^
miR-21	↑	Pitx2c [136]	↓	Pig ATP model, HL-1 cells	Upregulation in AF was correlated with decreased PITX2C protein. Its overexpression in vitro decreased mRNA and protein while inhibition with antimiR-21 had opposite effects.	[17, 18, 40, 44, 53, 56, 59, 74, 96–98, 136]
		SPRY1 [17] Spry1 [18]	↓	5 valvular AF, 5 SR patients, TG mice expressing Rac1, neonatal rat fibroblasts, in vivo inhibition in an ischemic rat/mice model.	Upregulation in LAA from AF patients and in an ischemic mice model was seen and correlated with increased fibrotic content and a decrease of SPRY1. Administration of Ang-II induced an increase of CTGF and miR-21 in cardiac fibroblasts while Spry1 decreased.. In vivo inhibition with antagomir-21 or a 15-mer-LNA based antimiR-21 suppressed the fibrotic response and prevented increased AF susceptibility.	
		STAT3^b^ [98]	↑	Sterile rat pericarditis model with ATP, in vivo inhibition in pericarditis rat, neonatal and adult rat atrial fibroblats	Pericarditis in rats increased AF susceptibility and fibrosis and upregulated IL1B, IL-6, TGFB, TNFa, STAT3 and miR-21. In vitro inhibition of miR-21 suppressed STAT3 phophorylation, Col1A1 and Col3A1 mRNA, while overexpression had opposite effects. In vivo inhibition with antagomir-21 decreased STAT3 phosphorylation, fibrosis and AF vulnerability,	
		Smad7 [97]	↓	Rabbit ATP model, in vivo forced expression in rabbits, rat cardiac fibroblasts	Upregulation of TGF-β1 mRNA and protein in ATP rabbits correlated with increased miR-21 and decreased Smad7. In vivo pre-treatment with miR-21 inhibitor restored Smad7 and prevented a decrease in collagen I/III mRNA and protein. MiR-21 directly targeted Smad7.	
mir-26a	↓	TRPC3 [86]	↑	VTP canine model with CHF, ATP goat model, canine and rat cardiac fibroblasts	Downregulation in isolated LA fibroblast form AF dogs correlated with increased TRPC3 protein. Its overexpression in vitro suppressed TRPC3 protein and fibroblast number, while inhibition had opposite effects. Administration of NFAT-blocker increased miR-26a/b in vitro. MiR-26a directly targeted TRPC3.	[16, 46, 68–70, 86]
miR-29b	↓	COL1A1, COL3A1, FBN [103]	↑	RA from 17 AF, 19 SR patients, VTP canine model with CHF, canine fibroblasts, in vivo inhibition in mice, human AF plasma samples	Downregulation was seen in RA tissue of AF patients and LA tissue and fibroblasts from VTP dogs. Plasma levels of AF patients were also lower. VTP dogs demonstrated increased COL1A1, COL3A1 and FBN in fibroblasts. MiR-29b inhibition in vitro with miR-29b sponge increased mRNA levels and protein of those ECM components while overexpression had opposite effects. In vivo inhibition with miR-29b sponge in mice increased atrial COL1A1 and tissue collagen content.	[30, 103]
miR-30a	↓	Snail 1[104]	↑	Rabbit ATP model, cardiac rat fibroblasts	Downregulation in ATP rabbits correlated with increased Snail1 and Periostin mRNA and protein levels. MiR-30a overexpression xin vitro suppressed snail1 and periostin mRNA and protein, while inhibition increased their expression. MiR-30a directly targeted Snail1.	[104, 137]
miR-133	↓	TGF-β1 [107]	↑	19 AF patients, with or without nicotine abuses, canine model with nicotine administration, canine atrial fibroblasts	Downregulation in dogs and canine fibroblasts correlated with increased nicotine concentration. Nicotine usage was also associated with downregulation in human RA. MiR-133 overexpression in vitro decreased TGF-β1 protein and collagen content, while inhibition increased TGF-1 and collagen content. Nicotine administration in vitro decreased miR-133. MiR-133 directly targeted TGF-β1.	[44, 46, 107, 137]
miR-146b	↑	TIMP-4[108]	↓	30 parAF, 17 SR patients, mice cardiac fibroblasts	Upregulation in AF correlated with decreased TIMP-4. MiR-146b in vitro overexpression decreased TIMP-4, which could be prevented by inhibition. A downregulation of TIMP-4 was associated with increased MMP-9 and collagen content. MiR-146b directly targeted TIMP-4.	[37, 44, 53, 108, 109]
miR-208a/b	↑	Thrap1, myostatin GATA4 [111]	↓	Mice pressure overload model, rat cardiomyocytes, TG and KO mice	MiR-208a KO mice developed spontaneous AF. TG mice overexpressing miR-208a had reduced Thrap1 and myostatin protein levels and hypertrophic growth while KO induced an increase. MiR-208a/b directly targeted Thrap1, myostatin and miR-208 targeted GATA4.	[31, 53, 58, 62, 71, 74, 111, 114]
miR-208a/b	↑	Sox5, Sox6 [58]	↓	4 permAF, 2 SR patients, human cardiac myocytes, HL-1 cells	Upregulation in AF correlated with decreased Sox5, Sox6 and increased Myh7. Overexpression of miR-208b in vitro suppressed Sox6 and increased Myh7. Overexpression of miR-208a in vitro suppressed Sox5 and moderately increased Myh7.	[31, 53, 58, 62, 71, 74, 111, 114]
miR-590	↓	TGF-βR2 [107]	↑	19 AF patients, with or without nicotine abuses, canine model with nicotine administration, canine atrial fibroblasts	Downregulation in dogs and canine fibroblasts correlated with increased nicotine concentration. Nicotine usage was also associated with downregulation in human RA. MiR-590 overexpression in vitro decreased TGF-βR2 protein and collagen content, while inhibition increased TGF-β2 and collagen content. Nicotine administration in vitro decreased miR-590. MiR-590 directly targeted TGF-βR2.	[107, 114]

^a^Studies reporting about the specific miRNA in AF. These include both explorative and functional studies in tissue and plasma and may present conflicting data regarding upregulation or downregulation of the miRNA

^b^Not a direct target of the miRNA

Abbreviations: *AF* atrial fibrillation, *Ang-II* angiotensin-II, *APD* action potential duration, *ATP* atrial tachypacing, *CHF* congestive heart failure, *CTGF* connective tissue growth factor, *FBN* fibrillin, *FKBP5* FK506 binding protein 5, *KO* knockout, *LA* left atrium, *MMP-9* Matrix metallopeptidase 9, *MVS* mitral valve stenosis, *NFAT* nuclear factor of activated T-cells, *permAF* permanent/chronic AF, *PITX2* paired-like homeodomain transcription factor 2, *RA* right atrium, *Smad7* decapentaplegic homologue 7, *SPRY1* sprouty RTK signalling antagonist 1 gene, *SR* sinus rhythm/controls, *STAT3* signal transducer and activator of transcription 3, *TG* transgenic, *TGF-β1* transforming growth factor-β1, *TGF-βR2* transforming growth factor β receptor type 2, *THRAP1* thyroid hormone-associated protein 1, *TIMP-4* Metalloproteinase inhibitor 4, *TRPC3* transient receptor potential canonical-3, *VTP* ventricular tachypacing

### Ion Channel Remodelling

Remodelling of ion channels occurs within hours after the initiation of AF and is characterized by a prominent downregulation of L-type Ca^2+^ (I_CaL_) current and transient outward current (I_to_) and by an upregulation of inward rectifier K^+^ current(I_K1_) and acetylcholine-dependent K^+^ current (I_KACh_). These changes shorten action potential duration (APD) and the effective refractory period (ERP). Subsequent shortening of the wavelength (the mathematical product of ERP and conduction velocity) then facilitates AF induction and perpetuation [50].

For example, neuronal nitric oxide synthase (nNOS), which is an upstream regulator of several ion channels, was described to be suppressed by miR-31 [51]. Reilley et al. [52] found LA-specific upregulation of miR-31 in AF patients, and a goat model of AF. MiR-31 promoted the decay of nNOS and altered the localization of nNOS by repression of dystrophin translation. MiR-31-5p hairpin inhibitor in human atrial myocytes restored nNOS and dystrophin protein levels and APD, which may contribute to the termination of AF. MiR-31 expression in AF has not been reported to be upregulated in the explorative studies so far, but a downregulation in AF patients was described by two studies [53, 54].

#### Calcium Channels

Decreased I_CaL_, which shortens action potential duration, may result from a decreased expression of the L-type voltage-dependent calcium channel subunit α1C (Cav1.2; CACNA1C) and the L-type voltage-dependent calcium channel subunit β1 and β1 (Cavβ1/2; CACNB1/2) [50]. Several miRNAs have been implicated to regulate Ca^2+^-channels (Table 3).

##### miR-328

MiR-328 was the highest upregulated miRNA after microarray analysis of LA tissue of AF patients and in a canine atrial tachypacing (ATP) model [45]. In vivo adenoviral overexpression of miR-328 in dogs and transgenic mice overexpressing miR-328, resulted in decreased Cav1.2, Cavβ1 and I_CaL._, shortening of the APD and enhanced AF susceptibility. Consequently, antagomir-328 reversed the condition in dogs and genetic knockdown with a miR-328 sponge in mice decreased AF susceptibility. Interestingly, miR-328 was consistently found to be lower in plasma of both AF and heart failure patients [30, 38, 39, 55]. However, other AF studies specifically mentioned no difference in miR-328 in plasma [37] or atrial tissue [56] or even described an increase in plasma [57].

##### miR-208a/b

Canon et al. [58] performed a microarray screen in 4 patients with AF and 2 without AF and identified miR-208a and miR-208b in particular as the most significantly increased miRNA in AF. In this study, miR-208a was not significantly associated with reduced CACNA1C mRNA levels or decreased ICaL density. Moreover, transfection with either miR-208a or miR-208b in HL-1 cells decreased Cav1.2 protein levels. Luciferase assay confirmed CACNA1C and CACNB2 as direct targets of miR-208a/b. The miR-208 family is cardiomyocyte specific and encoded by introns of the cardiac myosin heavy chain genes (MYH6 and MYH7). Besides their role in electrical remodelling, Canon et al. [58] and others suggested that miR-208a and miR-208b play a role in structural remodelling, as described below and in Table 4.

##### miR-21

MiR-21 was upregulated in RA cardiomyocytes from AF patients and correlated with decreased CACNA1C and CACNB2 levels [59]. In vitro overexpression of miR-21 decreased I_CaL_ density and luciferase assays confirmed miR-21 to target CACNA1C and CACNB2. This miRNA has extensively been studied for its involvement in the structural remodelling underlying AF, described below and in Table 4.

#### Potassium Channels

An increase in inward rectifier current I_K1_, is a prominent feature of AF electrical remodelling. Increased I_K1_ results from increased expression of Kir2.1 protein encoded by KCNJ2 and causes shortening of APD and hyperpolarization of the membrane potential, which may promote re-entry and stabilize atrial rotors [60, 61](Table 3).

##### miR-1

MiR-1 is a muscle-specific miRNA and the most abundantly expressed miRNA in both ventricles and atria [62]. MiR-1 was upregulated in a rabbit ATP model along with a decrease of KCNE1 and KCNB2 mRNA and protein, shortening of the atrial effective refractory period (AERP) and increase of AF susceptibility. In vivo upregulation of miR-1 through atrial injection of a recombinant lentivirus carrying miR-1, resulted in enhanced downregulation of KCNE1 and KCNB2 and, unlike what may be expected, caused an increase in delayed rectifier potassium current (I_Ks_) and AF susceptibility. Conversely, anti-miR-1 attenuated the decrease of KCNE1 and KCNB2, and decreased AF vulnerability. KCNE1 and KCNB2 were confirmed as direct targets of miR-1 with luciferase assays [63]. However, because human atrial myocytes have different electrophysiological properties compared to rabbits, upregulation of I_Ks_ is less likely to contribute to AF pathophysiology in patients [64] and others have reported contradicting results about the regulation of miR-1 in AF (Table 3). Most importantly, Girmatsion et al. [15] found greatly reduced levels of miR-1 in atrial tissue of AF patients, which corresponded to an upregulation of KCNJ2, the inward-rectifier potassium ion channel protein (Kir2.1) and I_K1_. Furthermore, reduced miR-1 levels and increased Kir2.1 could be induced by ex vivo tachypacing of human atrial slices [15].

MiR-1 has extensively been studied in the context of ventricular arrhythmogenesis and interestingly, an upregulation of ventricular miR-1 was reported in patients with coronary artery disease and in a mouse model of ischemia, where it also had pro-arrhythmogenic effects [65]. Moreover, Zhao et al. [66] suggested miR-1 in the ventricle to target the transcription factor Irx5, which regulates several ion channels and gap junction proteins, and Terentyev et al. [67] related miR-1 to calcium handling abnormalities in the failing heart. As electrical remodelling in AF is also characterized by a dysregulation of ion channels and calcium handling, this suggests that the effects of miR-1 in AF are much broader than currently described.

##### miR-26a/b

A downregulation of miR-26 in atria of AF patients was associated with increased KCNJ2 and Kir2.1 [68]. Two subsequent studies of the same study group including both canine and mice tachypacing models resulted in a decreased expression of miR-26b and an even stronger decrease of miR-26a in atrial tissue [68] and cardiac fibroblasts [16]. In vitro knockdown of miR-26a with an LNA-based antimiR in canine fibroblasts induced an increase in I_k1_, hyperpolarized the resting membrane potential and increased fibroblast proliferation [16]. In vivo inhibition of miR-26a in mice with antimiR-26a increased I_K1_ and promoted AF, whereas in vivo adenoviral overexpression of miR-26a decreased I_K1_ and damped AF vulnerability [68]. Luciferase assays confirmed miR-26 to directly target KCNJ2 [68]. Of note, besides these two reports from the same study group, other discovery studies are inconsistent about the up- or downregulation of miR-26 isoforms in atrial tissue [56, 69, 70].

##### miR-30d

Morishima et al. [71] performed a microarray screen to identify miRNAs involved in electrical remodelling and found miR-30d to be highly expressed in patients with persAF, corresponding to downregulation of KCNJ3 and Kir3.1. MiR-30d was found to directly target KCNJ3 by luciferase assays and in vitro transfection of miR-30d was associated with a downregulation of KCNJ3, Kir3.1 and I_KACh_. In patients with sustained AF, the acetylcholine-regulated K^+^-current (I_KACh_), which is an inward rectifier current carried by the Kir3.1 and Kir3.4 subunits, was found downregulated [72]. However, another study revealed constitutive activity of I_KACh_ alongside an increase in I_K1_, which may contribute to APD shortening and AF [73].

##### miR-499

MiR-499 was upregulated in a microarray screen of RA tissue of 4 AF patients with and 4 patients without AF. Correspondingly, there was a decrease of the small conductance Ca^2+^-activated potassium channel (SK3) [74]. Luciferase assay confirmed the encoding KCNN3 as a direct target of miR-499 and in vitro overexpression of miR-499 downregulated KCNN3 and SK3 while antimiR-499 upregulated SK3 levels. MiR-499 is a cardiomyocyte-enriched miRNA and circulating miR-499 has extensively been studied as a biomarker of myocardial infarction and HF [75]. Three explorative studies reported miR-499 to be upregulated in tissue of AF patients [53, 71, 74], but in other studies, miR-499 was downregulated [45, 58].

#### Sodium Channels

Aside from potassium current remodelling, sodium channel (I_Na_) density may also be reduced in AF [76, 77]. This is supported by the fact that loss-of-function mutations in the SCN5A gene, encoding a subunit of the cardiac voltage gated sodium channel Na_v_1.5, have been associated with familial AF [78]. Zhao et al. [79] reported upregulation of miR-192-5p in AF patients which corresponded to downregulation of SCN5A and Na_v_1.5 protein. In vitro overexpression of miR-192-5p decreased I_Na_ density. MiR-192-5p is thereby the only miRNA that has been associated with sodium channel remodelling in AF.

### Calcium Handling

High atrial rates during AF cause a Ca^2+^-overload and an imbalance in intracellular Ca^2+^ homeostasis which contributes to AF perpetuation. Increased diastolic leak of Ca^2+^ from the sarcoplasmic reticulum through the ryanodine receptor 2 (RYR2) promotes increased Na^+^/Ca^2+^ exchange through the Na^+^/Ca^2+^exchanger (NCX) [80–82]. This, in turn, depolarizes the cell membrane and thereby facilitates triggered activity [83–85]. In addition, abnormalities in cellular Ca^2+^ homeostasis may indirectly affect structural and electrical remodelling. For example, Ca^2+^-overload may activate the Ca^2+^-dependent calcineurin/nuclear factor of activated T cells (NFAT) system, which results in hypertrophy and fibrosis [16, 86].

#### miR-208b

Ca^2+^ from the cytosol is transported back into the sarcoplasmic reticulum through the sarcoplasmic reticulum Ca^2+^adenosine triphosphates type 2a (SERCA2a), which thereby influences sarcoplasmic and cytosolic Ca^2+^ concentration. Canon et al. [58] found an inverse correlation between an upregulation of miR-208b, but not of miR-208a, and a decrease in SERCA2 mRNA in atrial myocytes from AF and control patients. In vitro overexpression of miR-208b also decreased SERCA2 protein expression.

#### miR-106b-25 Cluster

Downregulation of the miR-106b-25 cluster was seen in parAF patients compared to controls and was associated with increased RYR2 protein expression. RyR2 mRNA expression was unchanged, suggestive of an inhibition of RYR2 translation by miR-106b-25. Luciferase assay confirmed miR-93, belonging to the cluster, to directly target RyR2. Moreover, spontaneous local and global sarcoplasmic reticulum Ca^2+^-releases were increased in miR-106b-25 knockout mice, resulting in higher AF susceptibility [87].

### Extracellular Matrix Remodelling

Atrial fibrosis is considered the hallmark of atrial structural remodelling in AF. Fibrosis may promote re-entry by conduction slowing, increased anisotropy or unidirectional conduction block [88, 89]. Increased collagen content was found in AF patients compared to controls [90, 91] and an altered composition of ECM proteins was related to AF progression [89, 92]. Fibroblasts form the most abundant cell type in cardiac tissue and may, under pathophysiological conditions, provide pro-fibrotic signalling, or differentiate into myofibroblasts which secrete ECM proteins. Fibroblasts may furthermore by interactions with cardiomyocytes affect excitability and thereby conduction velocity [88, 93]. Various pro-fibrotic signalling pathways are involved in atrial fibrosis, such as the renin-angiotensin-aldosterone pathway, transforming growth factor-β1 (TGF-β1) [94], connective tissue growth factor (CTGF) and platelet-derived growth factor (PDGF) [88]. Here, we present the most important miRNAs that have been implicated as regulators of atrial fibrosis in AF.

#### miR-21

Upregulation of miR-21, highly expressed in fibroblasts, has been associated with increased cardiac fibrosis, not limited to AF [17, 18, 95–98]. Several mechanisms have been proposed for the potential effect of miR-21 on fibrosis, but most attention has been paid to miR-21 repression of SPRY1 (Sprouty 1, RTK signalling antagonist 1). Sprouty-1 inhibits the extracellular signal-regulated kinases (ERK) signalling pathway, which promotes fibrosis [17, 18, 95]. Upregulation of miR-21 was associated with downregulation of SPRY1 in patients with valvular AF and in rats with myocardial ischemia [17, 18]. In vivo inhibition with antagomir-21 in mice or a 15-mer LNA-based antimiR-21 in rats suppressed fibrosis and AF [18]. However, in the heart failure field contradicting results about the role of miR-21 in fibrosis have been reported. Thum et al. [99] showed that inhibition of miR-21 by antagomir injection protected mice against cardiac fibrosis and attenuated cardiac dysfunction in response to thoracic aorta constriction (TAC). On the other hand, Patrick et al. [100] reported that neither genetic deletion of miR-21 nor inhibition with tiny LNA-based antimiRs altered cardiac fibrosis in response to various stresses, like TAC and MI. Contradicting findings may results from different effectiveness of the antimiR-21 and antagomir-21 chemistries, used in the different studies [101].

MiR-21 may also promote inflammation-associated atrial fibrosis through the phosphorylation of the transcription factor signal transducer and activator of transcription 3 (STAT3). Inhibition with antagomir-21 in rats with pericarditis and AF suppressed STAT3 phosphorylation, the expression of fibrosis-related genes and AF vulnerability. MiR-21 promotes STAT3 phosphorylation through targeting the protein inhibitor of activated STAT3 (PIAS3) in multiple myeloma cells [102]. Expression of miR-21 itself was also found to be positively regulated by phosphorylated STAT3 and may thus form a feedback loop. Moreover, cardiac fibroblasts stimulated with the cytokine interleukin-6 increased STAT3 phosphorylation and miR-21 expression. This pathway may therefore link atrial inflammation to fibrosis formation [98].

Another suggested signalling pathway of miR-21 in AF involves the downregulation of Smad7, which is an inhibitory Smad of the TGFβ-pathway. Loss of Smad7 upregulates collagen I and III. MiR-21 expression was significantly increased in ATP rabbits along with a decrease of Smad7. In vivo inhibition of miR-21 suppressed the decrease of Smad7 and the increase of collagen I/III. Luciferase assays validated Smad7 as a direct target of miR-21 [97].

#### miR-26a

MiR-26 may, aside from potassium channel regulation, also play a role in ECM formation. MiR-26a was downregulated in the LA from dogs with heart failure and AF and corresponded to an increase of the Ca^2+^-permeable transient receptor potential canonical-3 (TRPC3) protein. Increased TRPC3 in turn stimulated fibroblast proliferation, differentiation and activation [86]. Luciferase assays validated miR-26a to directly target TRPC3 and in vitro inhibition of miR-26a with an LNA-based antimiR increased TRPC3 protein expression and promoted fibroblast proliferation. Moreover, increased TRPC3 expression was positively correlated with ERK phosphorylation and the expression of several ECM-related genes. Expression of miR-26 itself may be under the control of the NFAT system. The NFAT system downregulates miR-26 in response to Ca^2+^-loading and promotes fibroblast proliferation [86].

#### miR-29b

MiR-29b was downregulated in the atrium and in fibroblasts from dogs after ventricular tachypacing [103]. Both AF duration (after burst pacing) and atrial fibroblast COL1A1, COL3A1 and FBN mRNA increased significantly after prolonged ventricular tachypacing in these dogs. Moreover, overexpression of miR-29b in canine fibroblasts decreased COL1A1, COL3A1 and FBN expression, along with a decrease of Col1a1 protein in the supernatant. Adeno-associated virus (AAV) mediated knockdown with a miR-29b sponge had the opposite effect in fibroblasts and increased atrial Col1a1 mRNA and ventricular collagen content (Masson trichrome) in AAV-mediated knockdown mice [103]. Interestingly, this study also found miR-29b levels to be lower in tissue and plasma of patients with heart failure and concomitant AF.

#### miR-30a

In a rabbit ATP model, miR-30a was decreased in atrial tissue along with an increase of the transcription factor Snail 1, the matricellular protein Periostin and fibrotic tissue [104]. A functional role of miR-30a in the regulation of Snail1 and Periostin was revealed by overexpression and inhibition of miR-30a in rat cardiac fibroblasts [104]. Luciferase assays confirmed Snail 1 as a direct target of miR-30a. However, the mechanisms by which Snail 1 regulates Periostin, remains unclear.

Moreover, Li et al. [105] also associated the downregulation of miR-30 and miR-133 with an increase in fibrosis (Masson’s trichrome) in dogs with AF induced by tachypacing. However, they did not elaborate on potential targets of these miRNAs. In the heart failure field, Duisters et al. [19] concluded that miR-30 and miR-133 directly target CTGF and thereby control collagen content. Of note, miR-30a was described upregulated in AF patients in two studies investigating the LAA [46, 106] and in one study in the RAA [71]. Downregulation was seen in two studies investigating the RAA [44, 46].

#### miR-133 and miR-590

The expression levels of miR-133 and miR-590 were decreased in human atrial tissue of AF patients with nicotine abuses compared to non-users, and in a canine model with nicotine administration [107]. Transfection of miR-133 and miR-590 in canine atrial fibroblasts decreased TGF-β1, TGF-βRII and collagen content, which was reversed by inhibition by antagomirs respectively. Nicotine administration induced a downregulation of miR-133 and miR-590 in fibroblasts in a dose-dependent manner. TGF-β1 and TGF-βRII were established as direct targets of miR-133 and miR-590, respectively. MiR-133 was previously described to directly targeted CTGF, an important pro-fibrotic protein [19]. Moreover, CTGF is induced by TGF-β1 [19]. In explorative studies, miR-133 was downregulated in RAA in AF patients [44, 46].

#### miR-146b

Wang et al. [108] used LAAs from patients with and without AF and performed an integrated analysis of miRNA and mRNA expression profiles using microarray discovery followed by qPCR validation. This resulted in the miRNA-mRNA pair: miR-146-5p and tissue inhibitor of metalloproteinase 4 (TIMP-4). TIMP-4 is thought to inhibit matrix metallopeptidase 9 (MMP-9), involved in the degradation of extracellular matrix and formation of fibrosis. MiR-146b-5p was upregulated in AF patients along with an increase in MMP-9 and collagen content, but downregulation of TIMP-4. TIMP-4 was established as a direct target of miR-146b-5p by luciferase assays and transfection of miR-146b-5p in cardiac fibroblasts reduced TIMP-4 and increased collagen content. MiR-146b expression was furthermore correlated with LA diameter, AF duration and high-sensitivity CRP plasma levels [109]. In one of the explorative studies, miR-146b was actually the most upregulated miRNA in AF [44].

#### miR-208a/b

MiR-208a and miR-208b have frequently been implicated in AF pathophysiology and were described above for their role in Ca^2+^-handling and calcium channel regulation. However, these miRNAs are much better known for their role in structural remodelling in cardiovascular disease. MiR-208a and miR-208b are located within an intron of the α-cardiac muscle myosin heavy chain (MYH6) and the β-cardiac muscle myosin heavy chain (MYH7) gene, respectively [110]. Callis et al. [111] found that transgenic mice overexpressing miR-208a developed cardiac hypertrophy with suppressed expression of the targets thyroid hormone-associated protein (Thrap1, a known repressor of MYH7 transcription) and myostatin (a known repressor of muscle growth) [112]. MiR-208a knockout mice, on the other hand, developed spontaneous AF, accompanied by decreased Connexin 40 and elevated GATA4 protein levels [111]. However, the mechanism of arrhyhtmogenesis induced by miR-208 depletion remained unclear.

Canon et al. [58] found increased expression of miR-208a and especially of miR-208b in tissue of AF patients. Computational analysis predicted Sox5 and Sox6, negative factors of Myh7 transcription, as putative targets of miR-208. In vitro overexpression of miR-208a and miR-208b suppressed the expression of Sox5 and Sox6, respectively. Because the atrial tissue of AF patients showed a drastically increase of MYH7 protein levels, it is suggested that the increased expression of miR-208a/b in AF contributes to high MYH7 protein levels via inhibiting the expression of Sox5/6. Given that the healthy adult heart mainly expresses miR-208a and not miR-208b, miR-208a may initially target Thrap1 in AF pathophysiology. This in turn promotes MYH7 and simultaneously miR-208b transcription, which targets Sox5/6 and ultimately reinforces MYH7 transcription. MYH7 is a hallmark of cardiac hypertrophy, and the switch in MYH6:MYH7 expression ratio is linked to cardiac hypertrophy and heart failure. However, the mechanistic implications of MYH7 in AF remain unclear. MiR-208 has extensively been studied and was suggested as diagnostic biomarker of acute myocardial infarction or as therapeutic target in HF. For example, inhibition of this miRNA in several HF models has successfully prevented the formation of both cardiomyocyte hypertrophy and fibrosis, as discussed below [28, 110, 113]. Altogether, numerous studies associated miR-208a and miR-208b expression levels to cardiovascular disease and AF and proposed pathophysiological mechanisms involved in AF electrical and structural remodelling [31, 53, 58, 71, 74, 111, 114]. MiR-208 should therefore be considered as a potential target for AF therapy.

### Autonomic Nervous System

Autonomic dysregulation plays an important role in AF onset and maintenance [115]. This involves the upregulation of the acetylcholine dependent Ik^+^ current (I_KACh_) which shortens APD [116]. Furthermore, structural remodelling of the autonomic nerves consists of sympathic hyperinnervation of the atria and an imbalance between sympathetic and parasympathetic nerves [117, 118].

No miRNA has directly been associated with autonomic dysregulation in AF. As described above, miR-30d was found upregulated in patients with persAF and associated with downregulation of I_KACh_ [71]. Another study in ATP dogs demonstrated highly increased miR-206 levels, associated with structural remodelling of the autonomic nerves. In vivo lentiviral mediated overexpression of miR-206 in ATP dogs was associated with increased reactive oxygen species (ROS), nerve density and shortened AERP [119]. Luciferase assays confirmed that miR-206 directly regulated the anti-oxidant superoxide dismutase 1 (SOD1). These results suggest that miR-206 may induce autonomic nerve remodelling through a decrease of SOD1 and an increase of ROS.

### Other miRNAs in Atrial Fibrillation

Some miRNAs found to be involved in AF pathophysiology have not been associated to the regulation of a specific ion channel or extracellular matrix genes. For example, the upregulation of miR-199a in AF was found to suppress and target FKBP5, also known as the FK506 binding protein 5, an immunoregulative protein. Chiang et al. [69] performed a miRNA-mRNA interaction study in atrial biopsies from patients with AF and matched controls. The upregulation of miR-199a correlated with a downregulation of FKBP5. Luciferase assays revealed a direct interaction between miR-199a-5p and FKBP5. FKBP5 may interact with heat shock protein and may be involved in stabilizing microtubules and intracellular trafficking. However, FKBP5 may also affect Ca^2+^-regulation, but its function in AF pathogenesis has not yet been demonstrated [120]. MiR-199a has been associated with POAF by Yamac et al. [32], who suggested miR-199a to target SIRT1, a protein with antioxidant activity.

## Discussion

### Limitations of microRNA Studies

Circulating and tissue miRNAs regulate determinants of AF pathophysiology and have emerged as biomarkers of this disease. In this review, we present an extensive list of supporting evidence for the role of miRNAs in AF, but the inconsistencies among the explorative and functional studies cannot be denied. So far, no distinct miRNA has been identified as a clinically useful biomarker or as target for AF treatment. The inconsistencies between studies might be the result of the variation between studies in biological and technical design and more standardized comparisons of different disease models and technical approaches for modulation of miRNA levels are needed before miRNAs can be used in clinical practice.

Many of the discrepancies between plasma and tissue studies may be the result of the biological variation between the populations studied. Study populations used for explorative studies were small and usually did not include over 10 samples per group whereas some studies were even based on pooled samples for the discovery phase. In general, study designs did not allow to take the progression of AF into account, although study populations ranged from parAF to permAF. Furthermore, study populations consisted of patients with various, but relevant comorbidities such as CABG or mitral valve disease, but frequently, studies provided inadequate clinical details or were insufficient in size to correct for these comorbidities. Finally, miRNA expression can be tissue specific and miRNA differences are likely to depend on the origin of the tissue which can be RA or LA. Indeed, studies investigating both RA and LA found significant differences in miRNA expression between these two atria [44, 46, 56, 106, 114].

From a technical point of view, variation between studies can be introduced at several levels. For example, at the time of sample retrieval, patients may have received heparin for cardiothoracic surgery or plasma may have been stored in heparin holding tubes. Heparin interferes with enzymes in the RT-PCR and thus may affect its results [121]. Furthermore, most studies used a diverse range of microarray platforms for the discovery phase. The limiting aspects of microarray technology such as low comparability and sensitivity may be in part responsible for the discrepancies between studies [122]. Data-analysis pipelines also showed clear variation in the thresholds used for miRNA selection and studies rarely performed multivariate analyses.

Studies usually validated the microarray discoveries with qPCR amplification. qPCR is the most sensitive technique to detect miRNAs, but reliability may be hampered by low miRNA levels, especially in plasma. Because of low miRNA levels, normalization is particularly important for plasma studies. This requires standardized techniques and an adequate correction for differences in starting material with reference genes (tissue) or reference miRNAs or miRNA spike-ins. The optimal endogenous reference panel may vary with the present clinical characteristics of each study population in both plasma and tissue. Tissue miRNA levels are generally high and normalization has not been problematic in other cardiovascular disease, but this may not be the case for AF as a recent report indeed demonstrated that the commonly used reference gene U6 was the worst normalizer of a panel of five for human atrial tissue [123]. To date, there is no standardized protocol for the use of endogenous normalizers in AF. Alternatively, synthetic miRNAs such as Caenorhabditis elegans-derived miRNAs have successfully been spiked-in as exogenous normalizers, but only when added after full inactivation of RNase activity [23].

The discovery of miRNA biomarkers in animal models may not directly be extrapolated to the diagnosis or prognosis of clinical AF. Firstly, human heart physiology differs from the animal heart and artificial AF models simplify the complexity and multifactorial character of the disease [64]. Animal models of AF used for miRNA studies enable functional analysis, but studies rarely started with an explorative miRNA expression screen to select the most differentially expressed miRNAs. MiRNAs studied for their function were usually selected based on an established mechanism in other (cardiovascular) diseases, or were derived from previous studies in AF.

Altogether, we can conclude that there are many factors contributing to the inconsistencies between both functional and explorative studies.

### Future Perspective of miRNAs as Circulating Biomarkers

Despite the inconsistencies of miRNA expression among the current studies, circulating miRNAs remain promising biomarkers of AF. However, the specificity, the origin and function of miRNAs in the circulation are largely unknown. MiRNAs in the circulation have been demonstrated in conjunction with apoptotic bodies, microvesicles, exosomes or the so called protein-protected protein-miRNA complexes [20, 23, 24]. Circulating miRNAs are suggested to play a role in cell-to-cell signalling [124, 125]. For example, Bang et al. [126] demonstrated that cardiac fibroblasts excreted miR-21-3p in exosomes, which induced hypertrophy in cardiomyocytes. However, most miRNAs are thought to be excreted with protein complexes and to have limited function in cell-to-cell signalling [127, 128].

Circulating miRNAs do not necessarily reflect miRNA tissue levels. In this study, we demonstrated many contradictions in atrial tissue and plasma levels of specific miRNAs. Furthermore, our results imply that in tissue of AF patients, miRNAs were more frequently upregulated, whereas miRNAs levels in plasma were more often lower in AF patients. For example, plasma miR-328 levels were described to be lower in prevalent AF in multiple studies [30, 38, 39], whereas tissue miR-328 expression increased in dogs with AF and in LA tissue of AF patients [45]. A possible explanation for the contradicting increases and decreases in tissue and plasma could be retaining of cellular miRNA at the expense of miRNA secretion into the circulation. Alternatively, it is intriguing to speculate that the uptake by affected cells of circulating regulator miRNAs to restore intracellular levels might contribute to the difference between tissue and plasma levels.

If possible, miRNA expression in AF should be studied in the different compartments of the blood separately and tissue and plasma levels should be studied in parallel for a better understanding of their relation and indications for the specificity of circulating miRNAs. Because AF is such a complex disease, future studies should ideally hold larger study populations and perform more detailed clinical profiling to enable analysis of comorbidities. Ideally, studies should not only focus on the diagnostic value of miRNAs for AF presence, but also prospectively investigate the prognostic value of a single miRNA or a panel of miRNAs, for the occurrence of new-onset AF, AF recurrence or AF progression. In addition, miRNAs may be studied for monitoring of the disease or response to AF therapy.

### Future Perspective of miRNAs as Therapeutics

MiRNAs hold a promise for the development of a new class of therapeutics as expression and function can be enhanced or repressed by the systemic or local delivery of synthetic miRNA mimics and inhibitors respectively [129]. MiRNA mimics are double-stranded oligonucleotides that resemble the miRNA-duplex. The ‘guide strand' from the miRNA mimic is then incorporated in the RNA-induced silencing complex (RISC) to become functional and bind to the target mRNA. As the ‘guide strand’ from the miRNA mimic has to be recognized properly and undergo processing as an endogenous pre-miRNA, chemical modification for the delivery of miRNA mimics to the site of function is more challenging compared to antimiR chemistry. MiRNA overexpression may therefore be established using different serotypes of AAVs to increase organ and cell specificity [130]. AAVs may also be used for knockdown with the delivery of a miRNA sponge. AntimiRs are antisense singe-stranded oligonucleotides that are complementary to a full or a part of the mature miRNA and block its function after hybridization. AntimiRs can be chemically modified at their sugar backbone to increase their stability, prevent against degradation and improve their cellular uptake (e.g. 2’-O-methoyethyl, 2′-fluoro, LNA modifications and cholesterol particles) [21, 131].

MiRNA research in AF and their role in atrial cardiomyopathies lags behind research in the field of ventricular cardiomyopathies since the first human explorative studies in AF were performed in 2011 and most functional studies, besides a few exceptions, date from 2012 onwards. Meanwhile, progress has been made on miRNA-based therapies in pre-clinical trials for other cardiac disease**s**. As an example, miR-208 is one of the most important miRNAs studied as a potential target for miRNA inhibition in ventricular cardiomyopathies [113]. It was also designated as an important regulator of atrial remodelling in this review and could thus also play a role in atrial cardiomyopathies. MiR-208a knockout mice exhibited reduced fibrosis in response to cardiac stress and failed to upregulate Myh7 [110]. Montgomery et al. [113] demonstrated that the delivery of an LNA-based antimiR against miR-208a suppressed fibrosis, improved cardiac function and improved survival in a hypertension induced heart-failure model in rats. Of note, caution with the inhibition of miR-208 should be taken as miR-208a knockout mice displayed spontaneous AF [111]. Meanwhile, no clinical trials have been performed with miRNA therapeutics in cardiovascular pathology, but in other fields, miRNA therapeutics have successfully shown to suppress hepatitis C virus replication in phase 2a clinical trials without long-term relevant side-effects [132].

Functional studies with in vivo manipulation of miRNAs in AF suggest that also for atrial cardiomyopathies, a specific miRNA based therapeutic might be developed [17, 45, 63]. For example, Lu et al. [45] found that after in vivo adenoviral mediated forced expression of miR-328, the antagomir-328 successfully reversed AF susceptibility in ATP dogs. Jia et al. [63] demonstrated the potential of inhibiting miR-1 by administration of LNA-based antimiR-1, which prolonged AERP and reduced AF susceptibility and duration in ATP rabbits.

Before miRNA-based therapy is implemented in clinical practice, concerns about the safety of miRNA therapeutics in humans need to be overcome [21, 131, 133]. The most important concern about miRNA therapy arises from its potential to target multiple pathways. MiRNAs may interfere with physiological pathways by the delivery of (a high load of) miRNA mimics in non-targeted organs, or non-targeted pathways in the targeted tissue. MiRNAs mimics could also interfere with normal gene regulation through competition with endogenous uptake of double-stranded RNA or compete with incorporation in the RISC complex. Conversely, miRNA inhibitors may also have off-target effects. Using tissue-specific miRNA inhibitors or targeted delivery of miRNA mimics or inhibitors, which should be the focus of future research, could overcome these issues. AAVs could be used for targeted delivery, but as these rely on the delivery of genetic material, the effects may be permanent and long-term negative effects need to be established [130, 134]. On the other hand, anti-miRs inhibit miRNA function for a prolonged period of time, but they are eventually degraded and thus their pharmacokinetic properties may be improved by chemical modifications. [113]. The first clinical trials with the systemic delivery of liver specific miRNAs did not show long-term adverse effects and miRNA therapeutics remain promising [132]. Meanwhile, future studies should focus on in vivo effects of cardiovascular miRNA therapeutics in order to demonstrate safety and firmly determine therapeutic potentials [21].

## Conclusion

In this systematic review, we present up-to-date evidence on the role of miRNAs in AF pathophysiology. Explorative studies have indicated tissue and plasma miRNAs to be differentially expressed in patients with and without AF and functional studies implicated miRNAs in several pathophysiological pathways. However, the controversy among the studies was striking and careful attention needs to be paid when interpreting previous studies about the discovery or function of miRNA(s) in AF. Despite the explicit variation among the studies investigating miRNAs in AF, they may help to uncover the mechanisms underlying AF, have the potential to form a new class of biomarkers and promote the development of innovative therapies.

## Electronic supplementary material

ESM 1 (DOC 57 kb)
