# Supplementary material for: MicroRNAs in Atrial Fibrillation: from Expression Signatures to Functional Implications
Source: Cardiovasc Drugs Ther. 2017 Jul 28;31(3):345–65. doi: 10.1007/s10557-017-6736-z (PMC5550535; doi:10.1007/s10557-017-6736-z)
Supplement: Supplementary file 1 — (DOC 57 kb) [file 10557_2017_6736_MOESM1_ESM.doc]

# MicroRNAs in Atrial Fibrillation: from Expression Signatures to Functional Implications.

# *Cardiovascular Drugs and Therapy.*

#

Nicoline W.E. van den Berg, MD*, Makiri Kawasaki, PhD*, Wouter R. Berger, MD*, Jolien Neefs, MD*, Eva Meulendijks, Bsc*, Anke J. Tijsen, PhD*, Joris R. De Groot, MD, PhD*

* Academic Medical Center of Amsterdam, Heart Center, Departments of Cardiology, Experimental Cardiology and Cardiothoracic Surgery, Amsterdam, The Netherlands

E-mail: j.r.degroot@amc.uva.nl

# Supplementary Material

# Supplementary Methods

# Search Strategy

A systematic search was performed in OVID MEDLINE (31-10-2016) to identify relevant articles. The search terms included synonyms for “atrium”, “atrial fibrillation” and “microRNAs”. No methodological filters were used. We cross-checked reference lists and citing articles for to identify additional relevant studies.

**Supplementary** table 1. Search Strategy

| **Search 31-10-2016** | **Hits** |
| --- | --- |
| (atria*[tiab] OR atrium[tiab] OR auricular[tiab]) AND (fibril*[tiab] OR flutter*[tiab]) | 56548 |
| (microRNA*[tiab] OR miR[tiab] OR miRs[tiab] OR miRNA*[tiab] OR "micro RNA"[tiab] OR miRNome[tiab]) | 61351 |
| **Combined Total** | 117 |

# Article screening and grouping of studies

We excluded review articles and articles that did not provide full-text in English. We grouped articles based on the study aims, study design, materials, and techniques incorporated. First, we selected prospective studies. Next, we selected studies that incorporated explorative techniques (e.g. microarray, sequencing, massive parallel signature sequencing, high throughput qPCR) to study the miRNA expression profiles in tissue and in plasma of patients with AF and without AF. Studies were included if the miRNA discovery phase was performed in at least four patients per group. Finally, we grouped functional studies. We excluded studies that merely described an association between miRNA and target levels and provide no functional evidence in gain- or loss of function models or evidence of specific binding with luciferase reporter assay. Two articles were excluded because of poor quality.

# Data extraction

From explorative studies, we extracted study population and size, relevant patient characteristics, body materials used and the techniques. From each study, we listed all miRNAs that were described to be differentially expressed after the explorative phase based on the criteria and thresholds incorporated by the individual studies.

From functional studies, two readers independently extracted the models and techniques used to provide evidence for a functional role of miRNAs in AF.

# Supplementary Results

**Supplementary** table 2. Studies investigating miRNA expression and the onset of postoperative AF.

| **Study** | **Study population** | **Study design** | **Outcome** | **Tissue type** | **Technique** | **Results** |
| --- | --- | --- | --- | --- | --- | --- |
| Yamac et al.  Cardiovasc pathology  2016 | CABG no AF (n=63) | Prospective cohort | POAF (n=20), SR (n=43) | RAA | qPCR: miR-199a, miR-195  WB of SIRT1 protein | **Downregulated in POAF cases:** miR-199a  **Upregulated in POAF cases**: SIRT1 |
| Harling et al. Eur. Journal cardiothoracic surg.  2016 | CABG no AF (n=34) | Prospective cohort | Microarray: POAF (n=11), SR (n=11)  POAF (n=13), SR (n=21) | RAA  Plasma  (24h pre-op, 2d and 4d) | Microarray  qPCR | **Downregulated in POAF cases:** 19a, 208a‡, 24-1*, 29b, 301a  **Upregulated in POAF cases**: 1202, 150, 223, 2861, 3141, 3679-5p, 4298, 483-5p‡, 572, 638, kshv-miR-K12-3 |
| Slagsvold et al.  Circ res. 2014 | CABG no AF randomized to RIPC (n=30) and no RIPC (n=30) | RCT | POAF (n=18), SR (n=19) | RAA | microarray (10 vs 10 before and after cross clamping)  qPCR | Upregulated after cross-clamping: miR-133a, miR-133b  miR-1 only in the controls  miR-338-3p only in RIPC |
| Krogstad et al. Scandinavian cardiovasc journal  2014 | CABG no AF randomized to RIPC (n=45) and no RIPC (n=47) | RCT | POAF (n=27), SR (n=65) | Plasma | Microarray  (10 randomly selected RIPC and 10 no-RIPC patients) | No differences in miRNA expression |

‡ most upregulated or downregulated miRNA

Abbreviations: AF, atrial fibrillation; AUC, area under curve; CABG, coronary artery bypass grafting; d, days; POAF, postoperative AF; RAA, right atrial appendage; qPCR, quantitative polymerase chain reaction; RCT, randomized controlled trial; RIPC, Remote ischemic preconditioning; SR, sinus rhythm/controls; WB, western blot

**Supplementary table 3. MiRNAs expressed in plasma from AF patients**

| **Upregulated miRNAs (prevalent AF)** | |
| --- | --- |
| Described in 2 studies (n=5) | 9, 152, 374a, 454, 664 |
| Described in only 1 study (n=15) | 19, 19a, 125a-5p, 146, 146a, 146b-5p, 148b, 221, 342-3p, 409-3p, 421, 589, 598, 634, 941 |
| **Downregulated miRNAs** | |
| Described in ≥3 studies (n=2) | 99b-5p, 150-5p; 328 (4 out of 6 studies) |
| Described in 2 studies (n=3) | 100-5p, 375, 432 |
| Described in only 1 study (n=33) | let-7b-5p, let-7c-5p, 1, 10b-5p, 16-2*, 21-5p, 24-3p, 28-5p, 29a-3p, 30c-5p, 122-5p, 125a-5p, 125b-5p, 126-3p, 145, 146a-5p, 148b-3p, 162, 199a-5p, 199b-5p, 221-3p, 222, 223-3p, 320b, 331-3p, 338-5p, 339-5p, 342-3p, 409-3p, 411-5p, 486-5p, 487b, 493, 493b, 766, 874, 4732-3p |
| **miRNA contradicting in up- and/or downregulation between studies** | |
| n=6 | 125a-5p, 146a-5p, 148b-3p, 221-3p, 342-3p, 409-3p |

**Supplementary table 4. MiRNAs expressed in tissue from AF patients**

| **Upregulated miRNAs** | |
| --- | --- |
| Described in 3 studies (n=9) | 15b-5p, 21-5p, 24, 30a-5p, 142-3p, 146b-5p, 208b, 223-3p, 499-5p |
| Described in 2 studies (n=28) | Let-7d-3p, 19b, 22, 27b-3p, 30b-3p, 30e-3p, 93, 101-3p, 106b, 125b-5p, 145-3p, 185, 187, 199a-5p, 199b-5p, 210, 324-5p, 337-5p, 486-5p, 494, 505, 574-5p, 652, 1181, 1202, 1290, 1973, 4298 |
| Described in only 1 study (n=172) | let-7a, let-7f , 1, 7, 15a, 16, 18a, 18b, 19a, 20b, 23a, 23b, 25, 26a-3p, 27a, 28-5p, 30d, 32, 34a, 95, 103, 106a, 125a-3p, 125a-5p, 127-3p, 129-3p, 130a, 130b, 134, 140-5p, 142-5p, 144, 145, 148b, 149-3p, 152, 155, 181a, 181a-5p, 181c, 184, 188-5p, 190, 193a-3p, 193b, 196b, 198, 199a-3p, 203, 212, 215, 216a, 216b, 217, 222-3p, 224, 301a, 301b, 302a, 302b, 320, 330-3p, 331-3p, 335, 361-5p, 362-5p, 363, 371-3p, 372, 375, 377, 381-5p, 423-5p, 424, 431, 449a, 450a, 451, 454, 455-5p, 466, 483-5p, 487a, 487b, 495, 500, 504, 508-3p, 509-5p, 511, 517a, 517c, 518b, 518f, 519b-3p, 520e, 522, 539, 542-5p, 545, 548d-5p, 574-3p, 579, 590-5p, 597, 602, 618, 630, 660, 671-3p, 671-5p, 758, 762, 874, 885-3p, 886-5p, 887, 888, 940, 1207-5p, 1224-5p, 1225-5p, 1244, 1281, 1305, 1307-3p, 1308, 1910-3p, 1915-3p, 1972, 2861, 3125, 3135b, 3141, 3178, 3195, 3196, 3197, 3591-3p, 3610, 3613-3p, 3648, 3656, 3679-5p, 3939, 3940-5p, 4257, 4280, 4281, 4284, 4291, 4299, 4306, 4443, 4459, 4463, 4466, 4484, 4485, 4486, 4488, 4492, 4497, 4505, 4508, 4530, 4534, 4687-3p, 4707-5p, 6753-5p, 6820-5p, 7843-5p |
| **Downregulated miRNAs** | |
| Described in 3 studies (n=3) | 125b-5p, 143-3p, 145-5p |
| Described in 2 studies (n=17) | 21-5p, 22-3p, 24-3p, 26b-5p, 30a-5p, 30b-5p, 30c-5p, 31, 99a-5p, 125a-5p, 133b, 149, 181a-5p, 181c, 181d, 331-3p, 4281 |
| Described in only 1 study (n=95) | let-7a-5p, let-7c, let-7f-5p, 1, 10a, 16-5p, 23a, 23b-3p, 23c, 25-3p, 26a-5p, 27a, 29-3p, 29a-3p, 29c, 30d-5p, 30e, 95, 99b, 100, 107, 128, 133a, 138, 139-5p, 151a-5p, 151b, 152, 181b, 191-5p, 193a-5p, 193b, 195-5p, 197, 200b, 203, 208a, 221-3p, 222-3p, 299-3p, 324-5p, 361-5p, 367, 374b, 378, 378a-3p, 378d, 429, 451a, 455-3p, 484, 486-5p, 490-3p, 490-5p, 497, 500, 501-5p, 550, 628-5p, 720, 874, 885-5p, 1227-5p, 1270, 1273g-3p, 1915-3p, 2861, 3196, 3656, 3665, 3960, 4286, 4324, 4442, 4454, 4466, 4497, 4516, 4530, 4690-5p, 4707-5p, 4734, 4787-5p, 4800-3p, 5096, 5100, 5190, 5787, 6090, 6125, 6775-5p, 6786-5p, 6791-5p, 7110-5p, 7704 |
| **miRNA contradicting in up- and/or downregulation between studies** | |
| n=40 | Let-7a-5p, let-7f-5p, 1, 16-5p, 21-5p, 22-3p, 23a, 23b-3p, 24-3p, 25-3p, 27a, 30a-5p, 30d-5p, 30e-3p, 95, 125a-5p, 125b-5p, 145-5p, 149-3p, 152, 181a-5p, 181c, 193b, 203, 222-3p, 324-5p, 331-3p, 361-5p, 486-5p, 500, 874, 1915-3p, 2861, 3196, 3656, 4281, 4466, 4497, 4530, 4707-5p |
